# Comparison of Oncological Outcomes between Transperitoneal and Retroperitoneal Approaches in Laparoscopic Nephroureterectomies for Upper Tract Urothelial Carcinoma

**DOI:** 10.3390/medicina60030421

**Published:** 2024-02-29

**Authors:** Taiyo Otoshi, Takeshi Yamasaki, Taisuke Matsue, Nao Yukimatsu, Minoru Kato, Yuichi Machida, Tomoaki Iwai, Katsuyuki Kuratsukuri, Junji Uchida

**Affiliations:** Department of Urology, Graduate School of Medicine, Osaka Metropolitan University, 1-4-3 Asahi-machi, Abeno-ku, Osaka 545-8585, Japan; x21655r@omu.ac.jp (T.O.);

**Keywords:** upper tract urothelial carcinoma, laparoscopic nephroureterectomy, transperitoneal approach

## Abstract

*Background and Objectives*: Our aim was to clarify the oncological outcomes of the two different approaches to laparoscopic nephroureterectomies (LNUs) in Japan, and to examine whether there were any significant differences between the transperitoneal approach and the retroperitoneal approach. *Materials and Methods*: We retrospectively evaluated patients who underwent an LNU for upper tract urothelial carcinoma (UTUC) from January 2013 to December 2022. We identified 52 patients who underwent a transperitoneal LNU (tLNU) and 93 who underwent a retroperitoneal LNU (rLNU). We adopted age, smoking, and pT-stage matching, and 43 patients were classified in each group. We investigated the time from surgery to recurrence (RFS: recurrence-free survival), the time to death (OS: overall survival), and the time to non-urothelial-tract recurrence-free survival (NUTRFS). A Cox regression analysis was performed to evaluate the risk factors that influenced recurrence. *Results*: There were no significant differences in the RFS, OS, and NUTRFS between the two matched groups. In the multivariate Cox regression analysis, the pT stage (pT3≥ vs. pT2≤) had an HR = 2.09 and a *p* = 0.01, and was an independent prognostic risk factor regarding cancer recurrence. *Conclusions*: There were no significant differences in the oncological outcomes between the tLNU and rLNU groups. It is suggested that the transperitoneal approach should be selected for LNUs.

## 1. Introduction

Upper tract urothelial carcinoma (UTUC) is a malignant tumor that is present in the ureter and renal pelvis, accounting for only 5% of urothelial carcinomas [1]. A radical nephroureterectomy (RNU) is the standard treatment for localized UTUC. A laparoscopic RNU (LNU) has become widely adopted since the first case was reported in 1991 [2], and the advantages of its perioperative results compared to open surgery have been reported [3,4,5]. According to the European Association of Urology guidelines, an LNU is the standard surgical treatment for pT2 UTUC or lower [6]. Bladder cuff resections have been generally performed using the open method (cLNU: conventional LNU), but in recent years, there have been reports of pure LNUs (pLNUs) in which the entire surgery is completed laparoscopically [7,8,9]. There have been many reports of a transperitoneal approach to pure LNUs. However, there have been few studies investigating whether the transperitoneal or retroperitoneal approach should be used for LNUs. Although retroperitoneal dissemination and port site recurrence due to pneumoperitoneum are rarely observed in laparoscopic surgery [1,10], it is unclear whether there is a difference in the risk of nonspecific recurrence according to the approach utilized. In a study of perioperative results, Liu et al. [11] reported that the postoperative hospital stay, as well as the time to first oral intake, was significantly longer when the transperitoneal approach was used. In addition, Ye et al. [12] indicated that the total operative time was significantly shorter and that the postoperative pain was significantly lower when the transperitoneal approach was used. However, there have been very few studies on the oncological outcomes. Kim et al. [13] reported that the retroperitoneal approach significantly prolonged the progression-free survival (PFS) compared to the transperitoneal approach, but there was no significant difference in the overall survival (OS). In this paper, our aim was to clarify the oncological outcomes of the two different approaches to LNUs in Japan. 

## 2. Materials and Methods

### 2.1. Patients

Patients who underwent an LNU for UTUC at Osaka Metropolitan University Hospital from January 2013 to December 2022 were studied retrospectively. Patients without UTUC and those with a history of a radical cystectomy were excluded. The age, gender, body mass index, smoking status, tumor location (right or left), total operative time, laparoscopic surgery time, estimated blood loss, pathological T stage, location of the metastasis, follow-up duration, and history of bladder cancer were obtained from electronic records. For LNUs, the selection of either the transperitoneal or retroperitoneal approach was determined based on the patient’s history of abdominal surgery and the judgment of the attending surgeon. Regarding ureterectomies, a bladder cuff resection was generally performed with an incision in the lower abdomen. The tLNU group was the group of patients in whom the transperitoneal approach was used for nephrectomy. The rLNU group was the group of patients in whom the retroperitoneal approach was used for nephrectomy, as well as for ureterectomy and partial cystectomy procedures. In other words, this was the group in which no procedures were performed using the transperitoneal approach. Either approach is generally acceptable for a ureterectomy or a partial cystectomy. Some patients underwent pLNUs by an expert surgeon who had performed at least 100 LNUs prior to the observed procedures herein. cLNUs were performed by less experienced operators and in cases with a retroperitoneal approach. In the cases presenting with adhesion, such as from a past abdominal operation, a cLNU procedure was also chosen for the transperitoneal approach.

Cystoscopies and CT scans were carried out every 3 months for 1 year after the operation as an examination for postoperative follow-up purposes, and the follow-up was carried out every 6 months thereafter if there was no recurrence.

The patients were classified into two groups: a transperitoneal LNU (tLNU) group and a retroperitoneal LNU (rLNU) group. Age, smoking status, and pT-stage matching was performed. A total of 86 patients who underwent a tLNU or rLNU were matched.

We investigated the time from surgery to recurrence (RFS: recurrence-free survival), the time to death (OS: overall survival), and the time of non-urothelial-tract recurrence-free survival (NUTRFS) from the date of the first non-urothelial local recurrence, distant recurrence, or death for any reason, whichever came first.

Univariate and multivariate analyses were performed on the following five items as the factors involved in recurrence: gender, age, smoking status, surgical approach (retroperitoneal vs. transperitoneal), and pathological T stage (pT3≥ vs. pT2≤).

### 2.2. Surgical Technique

The surgical procedure for a radical nephroureterectomy is as follows.

When following the transperitoneal approach, the patient is placed in the lateral decubitus position. First, a laparotomy is performed on the outer edge of the rectus abdominis muscle at the level of the umbilicus; then, 12 mm ports for the left and right operators and 5 mm ports for the assistant are inserted, and the operation is performed with four ports. For a right nephrectomy, a 5 mm port for liver elevation is also inserted below the xiphoid process. The nephrectomy is then performed as usual. Whether or not to remove the adrenal gland is determined on a case-by-case basis, but, if possible, the gland is preserved.

When following the retroperitoneal approach, the patient is placed in the same position as in the transperitoneal approach. A camera port is inserted under the costal arch of the midaxillary line, and then 12 mm left and right operating ports are inserted. Then, a 5 mm port for the assistant is inserted into the lower abdomen, and the surgery is performed with four ports.

A lymph node dissection is not performed based on the template, and the region including the enlarged lymph node is sampled only in cases in which a significant enlargement of the regional lymph node is recognized in the preoperative CT image.

There are two patterns of bladder cuff incisions. In the case of a cLNU, the patient’s body position is changed to the supine position. After that, an incision is made in the midline of the lower abdomen, and the lateral bladder fossa is opened. The ureters are peeled away along the ureterohypogastric fascia, exposing the bladder muscularis. The umbilical ligament may be transected if necessary. After the bladder muscularis is exposed, the operator enters the bladder lumen to locate the ureteral orifice and transects the ureter with a bladder cuff. The bladder is sutured with a double-layer suture, where the mucosa and the muscle layer, as well as the muscle layer and the serosa, are continuously sutured to confirm that there are no leakages.

The transperitoneal approach is used for all cases of a pLNU, with 1 or 2 additional 12 mm ports in the lower abdomen at 10–15 degrees head down of the lateral decubitus position. After that, the procedure is similar to a cLNU for laparotomy, and the bladder is repaired with two layers of continuous sutures.

### 2.3. Statistical Analysis

Continuous variables are reported as the median and range. The Mann–Whitney U test was used for comparisons between the two groups. Kaplan–Meier survival curves were drawn for the PFS, OS, and NUTRFS, and significant differences were obtained using the log-rank test. The Cox proportional hazards model was used to detect hazard ratios and confidence intervals for a multivariate analysis of the recurrence rates. We also used a multivariable Cox regression analysis to examine the prognostic factors for the PFS. A *p*-value of 0.05 was considered statistically significant. All the statistical analyses were performed using JMP 10.0.2. (SAS Institute Inc., Cary, NC, USA) and EZR ver1.62 (Saitama Medical Center, Jichi Medical University, Saitama, Japan), which is a graphical user interface for R version 4.20 (The R Foundation for Statistical Computing, Vienna, Austria) [14]. 

## 3. Results

The patient backgrounds are shown in Table 1. A total of 145 patients were studied, with 52 in the tLNU group and 93 in the rLNU group. The baseline characteristics were similar between the two groups, except for the laparoscopic surgery time and the history of bladder cancer. There were no significant differences in the total operative time between the two groups or in the operative blood loss. No major complications were found except for one case of sigmoid colon injury in the rLNU group. A history of bladder cancer was more frequent in the tLNU group than in the rLNU group. The median follow-up was 34 months (range, 0.4–108) in the tLNU group and 41.3 months (range, 0.1–120) in the rLNU group. Neoadjuvant chemotherapy was performed in three cases in the tLNU group and in one case in the rLNU group. As for the location of metastasis, bladder recurrence was the most common in both groups. Peritoneal dissemination was only observed in one case in the tLNU group.

After adjusting for age, smoking status, and pT stage, 43 patients who underwent a tLNU were matched with 43 rLNU patients, as shown in Table 1. Similarly, no significant differences were observed, except for in the laparoscopic surgery time and history of bladder cancer.

**Figure 1:**
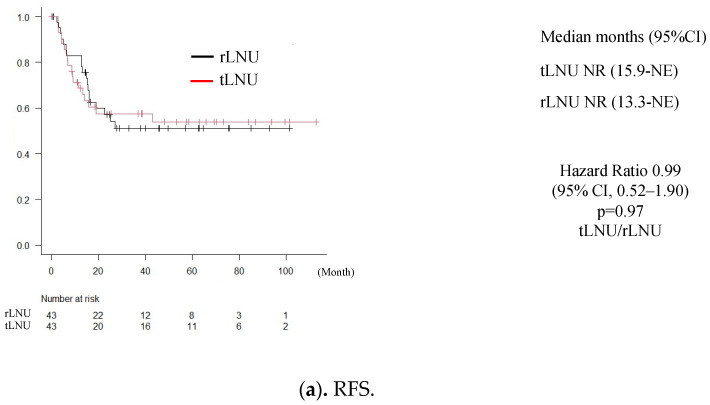

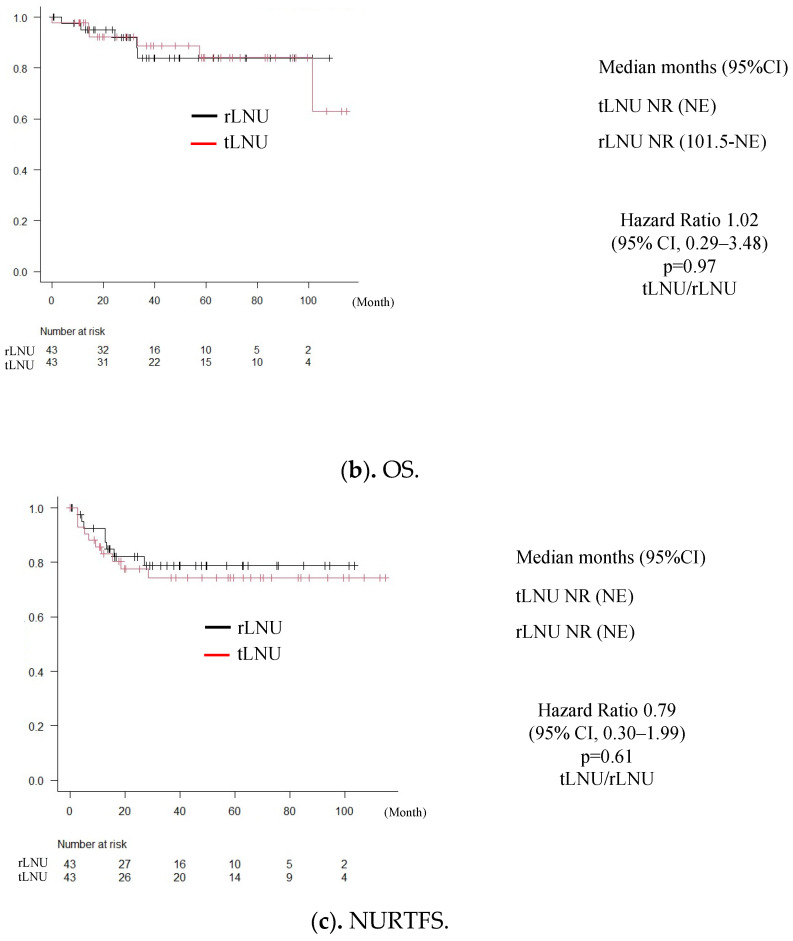
Cumulative recurrence-free survival, overall survival, and non-urothelial-tract recurrence-free survival of 86 matched patients treated with an LNU classified by the surgical approach (tLNU vs. rLNU).

Figure 1 shows the RFS, OS, and NUTRFS in the adjustment groups. With regard to the RFS, 19 patients (44%) in the tLNU group and 18 patients (42%) in the rLNU group had recurrences. The 2-year RFS rate was 62.8% in the tLNU group and 60.4% in the rLNU group, with no significant differences (HR = 0.99 (95% CI, 0.52–1.90), *p* = 0.98) (Figure 1a). With regard to the OS, there were five deaths (11.6%) in the tLNU group and six deaths (14.0%) in the rLNU group. The 2-year overall survival rate was 95.3% in the tLNU group and 90.6% in the rLNU group, with no significant differences (HR = 1.02 (95% CI, 0.29–3.48), *p* = 0.97) (Figure 1b). As for the NUTRFS, non-urothelial tract recurrence was seen in 8 cases (18.6%) in the tLNU group and 10 cases (23.2%) in the rLNU group, with no significant differences (HR = 0.61 (95% CI, 0.30–1.99), *p* = 0.61) (Figure 1c).

**Figure 2:**
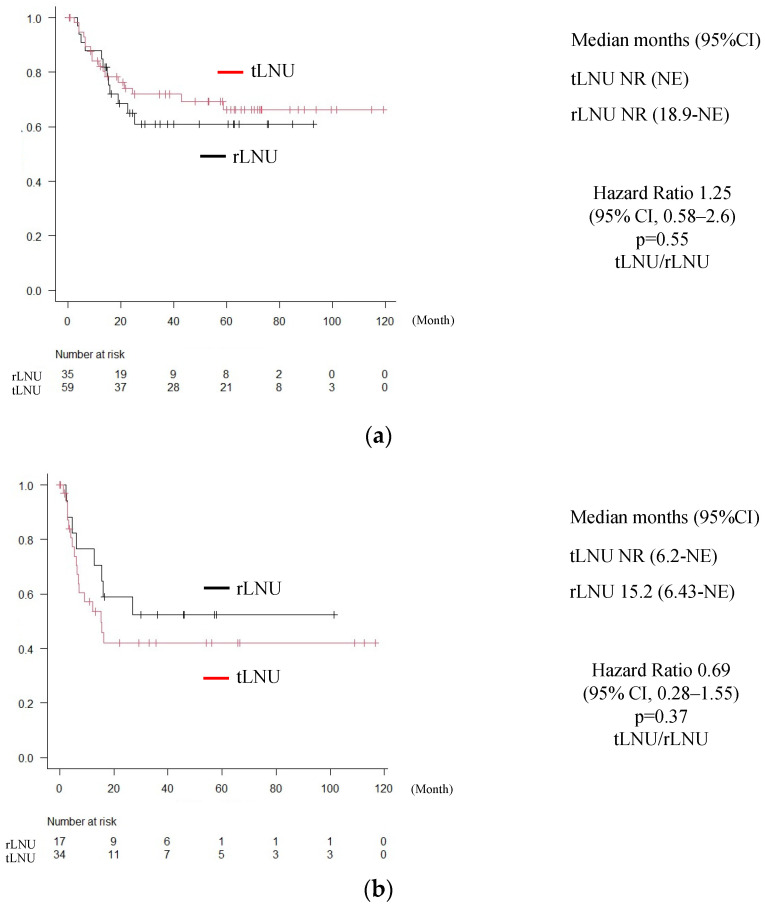
Recurrence-free survival classified by T stage. (**a**). Recurrence-free survival in patients with pT2 or lower. (**b**). Recurrence-free survival in patients with pT3 or higher.

We conducted a subgroup analysis, as shown in Figure 2. When the RFS in patients with pT2 or lower was examined, recurrence was found in 12 cases (out of 35 cases; 34.3%) in the tLNU group and 17 cases (out of 59 cases; 28.8%) in the rLNU group, with no significant differences (HR = 1.25 (95% CI, 0.58–2.60), *p* = 0.55) (Figure 2a). As for the RFS in patients with pT3 or pT4, recurrence occurred in 8 cases (out of 17 cases; 47.1%) in the tLNU group and 17 cases (out of 34 cases; 50.0%) in the rLNU group, with no significant differences (HR = 0.69 (95% CI, 0.28–1.55), *p* = 0.37) (Figure 2b).

We also investigated the factors involved in recurrence (Table 2). A univariate analysis of the RFS revealed a significant difference in the pT stage (pT3≥ vs. pT2≤). In the multivariate Cox regression analysis, the pT stage (pT3≥ vs. pT2≤) had an HR = 2.09 and was an independent prognostic risk factor. No significant differences were observed in the surgical approach (retroperitoneal vs. transperitoneal) (*p* = 0.91).

## 4. Discussion

In this study, we retrospectively compared the oncological outcomes of transperitoneal LNUs and retroperitoneal LNUs for UTUC in Japanese patients. In addition, matching was carried out for age, smoking status, and pT stage to more accurately compare both groups. 

The results showed that the oncological outcomes did not significantly differ between the two approaches. In addition, there were no significant differences in the RFS between the two approaches among patients with pT2 or lower, as well as among patients with pT3 or higher. The perioperative results were also examined, but no significant differences in blood loss or complications were found. Although there were no significant differences in the total operative time, the laparoscopic surgery time was significantly longer in the tLNU group. This was probably due to the higher percentage (64%) of patients undergoing a complete laparoscopic surgery (pLNU). The perioperative results were not significantly different between the two groups, except for in the laparoscopic surgery time, and there were also no differences in the oncological outcomes.

In recent years, the transperitoneal approach has been increasingly used due to the expansion of robotic surgery. Although the transperitoneal approach can provide a wide field of view, the leakage of urine containing urothelial cancer cells into the abdominal cavity might occur. Therefore, there has been concern that the risks of dissemination and recurrence are higher compared to the retroperitoneal approach. However, there have been very few reports on perioperative results and oncological outcomes according to the different approaches. 

Zhu et al. conducted a review of tLNUs and rLNUs [15]. The results showed that the tLNU group had a significantly shorter operative time, a significantly longer recovery time of intestinal function, and no significant differences in the estimated blood loss, length of stay, visual analog pain scale, or overall complication rates. Furthermore, no significant differences were observed in oncological outcomes such as the local recurrence rate, distant metastasis, and 1-year overall survival. The oncological outcomes were similar to those in our study. 

Wang et al. reported their findings on the use of the transperitoneal approach (28 patients) and retroperitoneal approach (12 patients) for pLNUs [16]. According to their results, blood loss was significantly higher with the transperitoneal approach. There were no significant differences in the operative time or other perioperative outcomes. The progression-free survival and mortality after 6 months were significantly worse with the transperitoneal approach.

Wu et al. reported a comparison of 94 tLNU cases and 172 rLNU cases [17]. The estimated blood loss in the tLNU group was significantly less than that in the rLNU group. No significant differences were observed in the 5-year overall survival, cancer-specific survival rate, intravesical recurrence-free survival rate, or disease-free survival rate. 

Liu et al. [11] reported the perioperative results for their transperitoneal and retroperitoneal groups, showing that the time to first oral intake was significantly longer and that the number of postoperative hospital days was significantly higher in the transperitoneal group, but other items were not significantly different. 

The highest number of cases was reported by Kim et al. [13]. They retrospectively reviewed and reported 743 cases of total nephroureterectomy for UTUC performed at a facility in South Korea and reported the oncological outcomes in the transperitoneal and retroperitoneal groups. There were 268 open surgeries and 475 laparoscopic surgeries, with 123 cases in the transperitoneal group and 620 cases in the retroperitoneal group. There were no significant differences in the 5-year cancer-specific survival or OS between the two groups, but the 5-year PFS was significantly lower in the transperitoneal group than in the retroperitoneal group. Similarly, in a subgroup analysis of 475 patients who underwent laparoscopic surgery, only the 5-year PFS was lower in the transperitoneal group. The authors speculated that this may have been due to the early ligation of the renal pedicle and ureter in the retroperitoneal approach, since the early ligation of the renal pedicle and ureter before manipulating the kidney or ureter may prevent the migration of tumor cells to the bloodstream or urinary system. However, in our study, there were no significant differences in the PFS or OS between the tLNU and rLNU groups. At our hospital, in both groups, the ureter was ligated immediately after processing the renal hilum at almost the same time, suggesting that the modification of surgical techniques may lead to a reduction in the recurrence rate. There were no significant differences between the two groups when factors involved in postoperative recurrence were examined. However, the multivariate analysis indicated that the pT stage influenced recurrence. 

Regarding postoperative intravesical recurrence, the preoperative bladder cancer complication rate was significantly higher in the tLNU group (tLNU, 28% vs. rLNU, 10%). However, there were no significant differences in postoperative bladder recurrence. Shigeta et al. [18] reported intravesical recurrence in 46% of patients after a total nephroureterectomy, while others have reported intravesical recurrence in 15–50% [19,20]. In our study, recurrence was reported in 25% of the patients in the tLNU group and 22% of the patients in the rLNU group, similar to the above reports. There were also no significant differences in the NUTRFS between the two groups. 

There has been concern that intraperitoneal dissemination may be increased when using the transperitoneal approach, but in this study, two local recurrences were observed in the tLNU group, with four in the rLNU group, and intraperitoneal dissemination was only observed in one case in the tLNU group. In recent years, it has been suggested that intraperitoneal dissemination can be prevented through appropriate management, even in transperitoneal groups, by using a bag when removing the tumor or using a closed circuit to prevent exposure to cancer tissues. In addition, the lower ureteral surgical process includes a procedure to open the bladder, and the possibility of urine containing urothelial cancer cells leaking into the abdominal cavity cannot be ruled out, but no local recurrences were observed in our study in spite of this. The influence of the surgical process is, therefore, thought to be minimal.

In our study, most cases of cLNUs were performed by surgeons with experience from 20–30 cases, but pLNUs were performed only by an expert surgeon who had experience from more than 100 cases. Shigeta et al. [7] reported their findings on the oncological outcomes of pLNUs and cLNUs. Their retrospective analysis using propensity score matching did not show significantly more recurrences in the pLNU group, but bladder recurrences were more frequent in the pLNU group in a subgroup analysis. Patients who underwent a pLNU had a significantly shorter operative time and less blood loss than those who underwent a cLNU.

As mentioned in our results, pLNUs resulted in a shorter surgical time and were superior to cLNUs from a cosmetic point of view, but they are technically difficult and not all surgeons can perform them. The bladder cuff incision technique of pLNUs requires familiarity with the development of the surgical field and the use of suturing techniques for bladder repair. Therefore, proficiency in laparoscopic surgery is required to some extent. The report by Shigeta et al. also indicates that pLNUs may have more intravesical recurrences. Dissemination may occur due to tumor compression during intraoperative manipulation. From this point of view, it is expected that the risk of seeding increases among less experienced operators.

If the operator’s experience is insufficient or the risk of tumor dissemination or complications is increased because of a previous abdominal surgery or cancer progression, a cLNU should be selected.

As mentioned above, according to past reports and this study comparing tLNUs and rLNUs, there are no significant differences in complications, and in many cases, there are also no significant differences in the oncological outcomes. The transperitoneal approach is not inferior to the retroperitoneal approach in terms of safety and cancer control because the operative space is wide and the operation can be carried out with the intestine and blood vessels in sight, even by operators who are unfamiliar with the laparoscopic operation. Additionally, as the number of robotic surgeries increases, more and more surgeons will become accustomed to the transperitoneal approach. This is because robotic surgery requires a wider operating cavity.

It seems that the use of the transperitoneal approach will also increase in laparoscopic surgery. We would be happy if this research could help in that direction.

The limitations of this study are that it was a retrospective study, and that the choice of approach was determined by the attending surgeon. Therefore, it is possible that there was selection bias, and that the patient background was not constant. In addition, many surgeons were involved, including those with less experience, meaning that the surgical techniques used were not uniform. 

In this study, there was a large number of surgeons with varying degrees of experience, which may have introduced bias. The surgeons ranged from those who had just started laparoscopic surgery to experts with experience from over 500 cases. In particular, a pLNU is a difficult procedure and is often performed by experts. Furthermore, the assistants included doctors with various years of experience, and it cannot be denied that this may have influenced the results.

However, with regard to laparoscopic nephrectomy, some reports have shown that the experience of the assistant does not affect the perioperative outcomes [21]. However, despite these limitations, there are few studies comparing the prognoses of the transperitoneal approach and the retroperitoneal approach in LNUs, especially in Japan. To our knowledge, this is the first such report in Japan, which we believe makes a very valuable contribution.

## 5. Conclusions

There were no differences in complications between the transperitoneal approach and the retroperitoneal approach in laparoscopic nephroureterectomies at our institution, and there were no significant differences in the perioperative outcomes, except for in the laparoscopic time. Furthermore, as in some previous reports, there were no significant differences between the two groups in terms of the overall survival, recurrence-free survival, and non-urothelial-tract recurrence-free survival. When we compared the recurrence-free survival of each group for pT2 or lower and pT3 or higher, no significant differences were observed.

Although this was not a prospective randomized controlled trial and the sample size was small, the results are comparable to those of previous reports. Further studies are needed in the future.

## Figures and Tables

**Table 1:** Characteristics of upper tract urothelial carcinoma patients who underwent transperitoneal or retroperitoneal LNU.

	tLNU (n = 52)		rLNU (n = 93)		*p*-Value
Age, years	76 (53–89)		75 (43–88)		N.S.
Gender, male/female	34/18		66/27		N.S.
Body mass index, kg/m^2^	23.0 (16.4–42.1)		21.9 (12.7–33.7)		
Smoking (%)	20 (38%)		43 (46%)		
Tumor location					
Renal pelvis	21		54		
Ureter	29		38		
Renal pelvis + ureter	2		1		
Right/left	19/33		43/50		
Neoadjuvant chemotherapy	3		1		
Operative time, min	294 (148–731)		291 (143–490)		
Laparoscopic surgery time, min	211 (106–402)		170 (50–314)		<0.001
Estimated blood loss, mL	50 (5–950)		75 (5–720)		
pT stage					
pT0, pTis, pTa, pT1, pT2	0, 3, 12, 11, 7	33 (63%)	1, 15, 12, 18, 12	58 (62%)	
pT3, pT4	18, 1	19 (27%)	31, 4	35 (38%)	N.S.
Location of metastasis					
Lymph node	1		8		
Liver	3		2		
Bone	1		2		
Lung	4		6		
Bladder	13		20		N.S.
Intrapelvic	2		4		
Residual ureter	1		1		
Adjuvant chemotherapy	7		10		
Months after surgery	34 (0.4–108)		41.3 (0.1–120)		N.S.
History of bladder cancer	12		9		0.03
	**tLNU (n = 43)**		**rLNU (n = 43)**		***p*** **-Value**
Age, years	76 (53–87)		75 (54–86)		N.S.
Gender, male/female	27/16		31/12		N.S.
Body mass index, kg/m^2^	23.0 (16.4–42.1)		21.9 (12.7–31.6)		
Smoking (%)	17 (40%)		17 (40%)		
Tumor location					
Renal pelvis	21		54		
Ureter	29		38		
Renal pelvis + ureter	2		1		
Right/left	17/26		20/23		
Neoadjuvant chemotherapy	3		1		
Operative time, min	279 (148–731)		293 (143–490)		
Laparoscopic surgery time, min	200 (105–370)		170 (79–314)		<0.001
Estimated blood loss, mL	45 (5–950)		50 (5–720)		
pT stage					
pT0, pTis, pTa, pT1, pT2	0, 3, 11, 10, 7	31 (72%)	1, 8, 6, 11, 6	32 (74%)	
pT3, pT4	11, 1	12 (28%)	9, 2	11 (26%)	N.S.
Location of metastasis					
Lymph node	1		5		
Liver	2		1		
Bone	1		1		
Lung	3		2		
Bladder	12		11		N.S.
Intrapelvic	2		1		
Residual ureter	1		1		
Adjuvant chemotherapy	5		4		
Months after surgery	33.3 (0.4–108)		42.8 (0.1–115)		N.S.
History of bladder cancer	11		3		0.02
N.S.: not significant					

**Table 2:** Univariate and multivariate logistic regression analyses for detecting risk factors of disease recurrence.

	Univariate			Multivariate		
	HR	95%CI	*p*-Value	HR	95%CI	*p*-Value
Female vs. male	1.35	0.76–2.33	0.28			
Age ≥ 75 vs. <75	1.02	0.98–1.05	0.21			
Smoking	1.21	0.49–1.43	0.5			
Surgical approach (retro- vs. transperitoneal)	1.03	0.599–1.83	0.91			
pT3≥ vs. pT2≤	2.09	1.21–3.56	0.01	2.31	1.33–3.99	0.01

## Data Availability

The data presented in this study are available from the corresponding author upon request.
